# A Study on Exfoliation of Expanded Graphite Stacks in Candelilla Wax

**DOI:** 10.3390/ma12162530

**Published:** 2019-08-08

**Authors:** Francesca Lionetto, Roberto López-Muñoz, Carlos Espinoza-González, Ricardo Mis-Fernández, Oliverio Rodríguez-Fernández, Alfonso Maffezzoli

**Affiliations:** 1 Department of Mathematics and Physics “Ennio De Giorgi”, University of Salento, Via per Arnesano, 73100 Lecce, Italy; 2 Department of Advanced Materials, Research Center for Applied Chemistry (CIQA), Blvd. Enrique Reyna 140, Saltillo 25294, Mexico; 3 Applied Physics Department, CINVESTAV-IPN, Apdo. Postal 73, Mérida 97310, Yucatán, Mexico; 4 Department of Engineering for Innovation, University of Salento, Via per Monteroni, 73100 Lecce, Italy

**Keywords:** candelilla wax, paraffin wax, graphite, exfoliation, rheology, dynamic mechanical analysis, sound velocity, attenuation, crystallization, nanocomposite

## Abstract

A novel, green route for pre-exfoliation of graphite based on a biodegradable polymer and high-power ultrasound is presented. Candelilla wax (CW), derived from the leaves of the candelilla plant, has been used for the first time as a natural non aqueous medium to induce the pre-exfoliation of expanded graphite (EG) under ultrasonic irradiation in an economical way. The proposed method uses also D-limonene as a natural organic solvent for reducing viscosity and increasing the affinity between the polar groups of EG and candelilla wax, thus improving the intercalation/exfoliation of EG. The quality of dispersion of the nanofiller in the natural wax matrix has been evaluated using multiple techniques. The addition of EG to wax and use of ultrasonic treatment leads to a reduced crystallinity, probably due to restrictions of the molecular movements, improved thermal stability of wax, and to an increased shear thinning exponent, which are all indicative of a high degree of EG dispersion. The ultrasonic dynamic mechanical results suggest a reduction in the cluster size and a better filler dispersion in the wax matrix promoted by polar or chemical reactions between the CW fractions and the graphite stacks, which was observed by XPS analysis. The results were compared to those obtained with paraffin, a synthetic wax, and confirmed the dispersion improvement obtained by using natural wax as a pre-exfoliating medium.

## 1. Introduction

**Figure 1:**
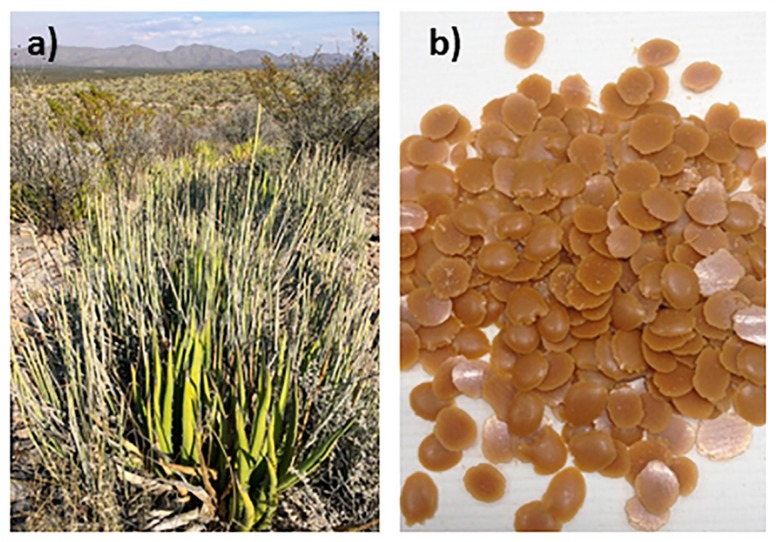
(**a**) Candelilla wild plant (*Euphorbia antisyphilitica*) grown in the northern Chihuahuan Desert; (**b**) candelilla wax pearls obtained after the extraction process in aqueous medium.

In recent years, the great concern of environmental pollution, has led to great interest in biodegradable polymers that are synthesized entirely or partially from annually renewable resources [1]. Among these, natural waxes are emerging materials that have continuously increased in their application possibilities. Natural wax is a mixture of long-chain, non-polar compounds, including hydrocarbons, waxy esters, sterol esters, ketones, aldehydes, fatty alcohols, and fatty acids [2]. Natural waxes are present on the outermost layer of plant surfaces, and they have the function to protect the plant from the loss of water and attack from insects [3]. Candelilla wax (CW) is a wax derived from the leaves of the candelilla plant (*Euphorbia antisyphilitica*), a shrub native to the Chihuahuan Desert region, an area encompassing northeastern Mexico and southeastern United States [2,3,4] (Figure 1). To survive the harsh environmental conditions, this plant secretes a thick wax layer. Candelilla wax has a melting point in the range of 69–73 °C, with chemical compositions mainly formed by odd-numbered *n*-alkanes (C_29_ to C_33_) that include wax esters, acid esters, and secondary alcohols, and chemical compositions in even-numbered carbon chains (C_28_ to C_34_) include free acids, free alcohols, sterols, resins, and minerals, which provide an outstanding balance between hydrophobic and hydrophilic properties [4,5,6].

CW has been industrially exploited ever since the beginning of the 1990s in cosmetic, food, pharmaceutical, and paint industries [2]. CW is a worldwide recognized food additive approved by the Food and Drug Administration (FDA) [5]. Recently, CW has been also used to prepare organogels, which are bi-continuous colloidal systems that coexist as a micro-heterogeneous solid that can immobilize an organic liquid phase resulting in a system with viscoelastic and thermo-reversible properties [5,7]. Until now, the studies on organogels based on CW have been related to food applications, but potential applications can also be envisaged in pharmaceutical, cosmetic, industrial, and art fields.

The availability and low cost of CW and its biodegradable nature has attracted growing interest from researchers towards the development of biocomposites that, thanks to their sustainability, energy efficiency, reduced waste generation, and low greenhouse gas emissions, are emerging powerfully in the current industrial economy [8,9,10,11]. Kowalczyk et al. [12] used CW to produce edible films and coatings, while Navarro-Guajardo et al. [4] proposed CW as a slow-release matrix for fertilizers encapsulated by a modified spray chilling process. Natural extracts, which are very commonly used in pharmaceutical, cosmetic, and food industries, can be used for improving the properties of candelilla, as already reported in the literature for many other biopolymers [13].

Potentially, CW can replace synthetic wax, such as paraffin wax, in countless applications, even as phase-change materials (PCMs), which are currently studied for solar energy storage, building energy savings, and temperature control in electronic equipment energy storage and thermo-optical switching [14,15,16,17]. PCMs, in particular those based on paraffin wax (PW), provide exciting applications only limited by the low thermal conductivity of PW [14]. This limitation can be enhanced with several methods, as proved by recent literature. Very recently, carbon-based materials have been investigated as attractive constituents to enhance the heat transfer of paraffin-based PCMs [18]. The most commonly used carbon material to improve PCM’s properties is expanded graphite (EG) [19]. EG is made from natural graphite flakes, which expand rapidly under high temperatures. It has a porous and accordion-like structure composed of hundreds of stacks of graphene nanosheets with an enormous surface area, good adsorption properties, high stability, and high thermal conductivity [20,21]. EG is an attractive filler for composites with various matrices and for colloidal suspensions of nanoparticles, which are extensively studied in the field of nanoscience and nanotechnology [22,23]. Pre-exfoliation of graphite is the most economical way to achieve large quantities of nanographite stacks, and a lot of methods for achieving exfoliation of graphite have been developed, each with advantages and disadvantages [24]. Among these methods, ultrasonic treatment with high-intensity ultrasound (already applied in a variety of industrial processes [25,26]) represents a powerful, safe, and environmentally friendly technique for dispersing carbon nanofillers by means of the shear forces coming from cavitation phenomena [27,28,29,30,31].

Polymer matrix nanocomposites attract a lot of attention since a small amount of filler can produce strong changes in the polymer with limited changes to their processability [32]. Many studies in the literature report the use of nanographite stacks to modify the functional and/or mechanical properties of polymers, often using dispersions in solvents [33,34,35,36]. The use of masterbatches has been an economic strategy to disperse nanographite stacks into polymers, since pre-exfoliation of graphite can be reached. However, preparation of masterbatches based on biodegradable matrices represents a significant challenge because of the hydrophilic nature of most matrices and their subsequent difficulty to host hydrophobic fillers, as widely reported in the literature [37]. Candelilla wax, to the best of our knowledge, has never been used as a masterbatch capable of promoting exfoliation of graphite stacks in polymers.

In this work, a novel, green route for the pre-exfoliation of graphite is presented based on biodegradable oligomers and high-power ultrasound. Candelilla wax has been used for the first time as an exfoliation medium of expanded graphite coupled with D-limonene. This organic solvent, derived from renewable sources, has been proposed with the aim of reducing viscosity and increasing the polar affinity between the polar groups of expanded graphite and candelilla wax, thus improving the intercalation/exfoliation of EG. The dispersion of EG in CW has been studied by means of multiple techniques. Shear rheology in the molten state, a widely recognized tool for correlating the rheological behavior of the nanostructure developed during processing [38,39], has been combined with thermal analysis, X-ray photoelectron spectroscopy (XPS), Raman spectroscopy, X-ray diffraction, and ultrasonic dynamic mechanical analyses. This latter technique has been recently proved to be very sensitive both to the complex molecular architectures in polymer matrices and to the scattering arising from filler agglomerates [28]. The results of EG–CW systems have been compared with those obtained on corresponding EG mixtures based on paraffin, a synthetic wax.

## 2. Materials and Methods

### 2.1. Materials

Candelilla wax (CW) was provided by Multiceras S.A de C.V (Monterrey, Mexico) with the trade name 7806 Candellila REAL. It was obtained from the plant *Euphorbia antisiphylitica*. As reported in the technical datasheet, its composition is mainly based on hydrocarbons (50%–57%), esters (28%–29%), alcohols, sterols and resins (12%–14%), and free acids (7%–9%), having an average molecular weight of 110–1641 g/mol. The investigated paraffin wax (PW)was 1061 Paraffin, provided by Multiceras S.A de C.V. It was made of linear hydrocarbons with an average molecular weight of 360–420 g/mol.

Expanded graphite (EG) was obtained in the laboratory starting from graphite intercalate compound (GIC) flakes with an average particle size of 300 µm supplied by Asbury Carbons (New Jersey, NJ, USA). D-limonene, purchased from ESENCITRICOS México S. A (Mexico City, Mexico), is a hydrocarbon classified as a monoterpene, commonly used as a solvent for organic compounds, alcohols, esters, and others, and as an additive in polymer matrices.

### 2.2. Preparation of Wax–Expanded Graphite (EG) Composites

**Figure 2:**
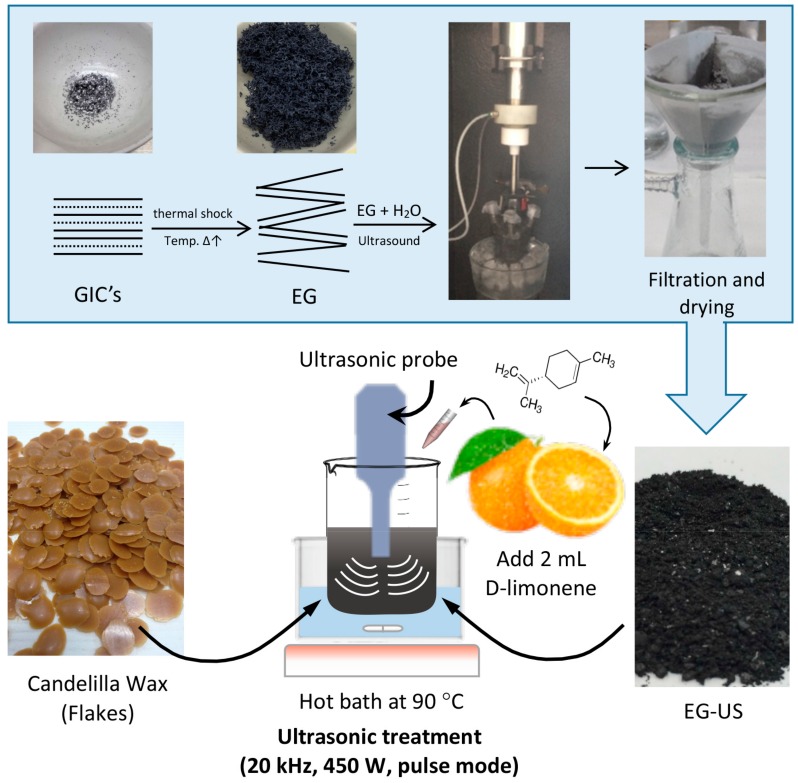
Scheme of the preparation of expanded graphite (EG)–wax nanocomposites. GIC, graphite intercalate compound.

The procedure for the preparation of EG–wax nanocomposites consisted of several steps summarized in the scheme shown in Figure 2. First, GIC flakes were expanded in an oven at 800 °C for180 s. Expanded graphite (EG) was thus obtained, in which polar groups such as –OH and –COOH were grafted after thermal treatment (for brevity, not reported). The expanded graphite (EG) was water dispersed by adding 1 g of EG into 150 mL of distilled water, which was sonicated for1 h at 420 W in a Q700 Sonicator (QSonica L.C.C, Newtown, CT, USA) with a power rating of 700 W and frequency of 20 kHz. Then, the solution was filtered and dried at 80 °C for 24 h (EG-US).

The EG–PW and EG–CW composites with an EG content of 10% by weight were initially prepared in the solid state in a conical reactor. The conical reactor was transferred into a hot bath with ethylene glycol at 90 °C until wax was completely molten. After addition of D-limonene (14% by weight), the mixture was subjected to an ultrasonic treatment with a Q700 Sonicator (QSonica L.C.C, Newtown, CT, USA) at 20 kHz and 450 W for 30 min. Ultrasonic irradiation was performed using a pulse mode (ON/OFF) because continuous irradiation in the batch process generates a rapid increase in temperature. A preliminary test allowed us to find the optimal conditions for pulse mode in order to maintain the temperature of the mixture at 90 °C. For paraffin wax compounds, the ON time was 55 s and the OFF time was 35 s. For candelilla wax compounds, the ON time was 30 s and the OFF time was 50 s.

Different organic solvents have been used to prepare graphene dispersions using ultrasonic energy [40]. Here, an organic solvent was added to melt wax as a strategy to reduce viscosity of the melt and attenuate the applied ultrasonic energy. It is known that acoustic cavitation and its effects on dispersion of nanostructures is easily induced in low-viscosity liquids [41]. The choice of D-limonene for graphite exfoliation was based on several criteria. First, D-limonene is a convenient high-boiling aromatic, biodegradable, and soluble in synthetic and natural waxes. Second, D-limonene is an aromatic molecule that must be able to interact with graphene via π–π stacking. Third, A yán-Varela and coworkers suggested that good solvents for exfoliation of graphite with oxidized carbon atoms in the edge-plane configuration, such as expanded graphite, are those with surface tensions approximately between 20 and 35 mJ m^−2^ [42]. D-limonene´s surface tension is 26 mJ m^−2^ [43], which is within the proposed range (20–35 mJ m^−2^).

The parameters of ultrasonic treatment (sonication time and power amplitude) were previously optimized. A power amplitude of 30%of the maximum and a sonication time of 30 min were chosen in this study. Temperature control during the ultrasonic treatment was kept stable with a warm bath at 55 °C.

For comparison purposes, some EG–PW and EG–CW composites were prepared only by magnetic stirring at 90°C for 30 min. These samples were labeled as EG–PW stirred and EG–CW stirred, respectively.

### 2.3. Characterization Methods

Raman characterization of graphitic materials was performed using a confocal microscope, Horiba Scientific model Xplora. The wavelength irradiation was 532 nm, 25% filter by 30 s of acquisition/accumulation in an interval test from 500 to 4000 cm^−1^.

Differential scanning calorimetry (DSC) was performed under nitrogen gas using a DSC discovery series from TA instruments (New Castle, DE, USA). Each sample was subjected to initial heating from −20 °C to 120 °C at 10 °C/min, then to a cooling cycle from 120 °C to −20 °C at 10°C/min and a second heating from−20 °C to 120 °C at 10 °C/min. In order to eliminate the effect of thermal history, results from DSC analysis on heating were collected during the second heating cycle.

The thermal stability of the samples was studied by thermogravimetric analysis (TGA). The samples were tested by using a TGA discovery series from TA instruments (New Castle, DE, USA) at a scanning rate of 10 °C/min from 25 to 600 °C in a nitrogen atmosphere and from 600 to 800 °C in an oxidative atmosphere.

The morphology of the samples was characterized by scanning electron microscopy (SEM) using a JCM-6000 NeoScope Benchtop (JEOL Inc., Peabody, MA, USA). The samples were subjected to a cryogenic fracture, and a layer of gold–palladium was deposited on the fracture surface.

The exfoliation of EG and wax composites was investigated by X-ray diffraction (XRD using a Rigaku Ultima IV (The Woodlands, TX, USA) with CuKα radiation at a scanning rate of 4.0 °/min using a voltage of 40 kV and a current of 44 mA.

Rheological analysis was carried out by using a parallel plate ARES rheometer from TA instruments (New Castle, DE, USA) with two parallel plates 25 mm in diameter. In order to determine the linear viscoelastic regime, dynamic strain sweep measurements at 80 °C and angular frequencies at 6.28 rad/s with strain ranging between 1% and 100% were performed. Then, frequency sweeps between 0.01 and 100 rad/s were applied over a linear strain (0.025%) at 80 °C. Specimens were placed between the preheated plates at 80 °C and were allowed to equilibrate for 10 min prior to each frequency sweep run.

Ultrasonic dynamic mechanical analysis (UDMA) was carried out on custom made equipment developed at the University of Salento [28]. Samples were loaded between two preheated ultrasonic transducers at 80 °C and were allowed to equilibrate for 10 min prior to each test. Then, ultrasonic measurements at 2 MHz were carried out during cooling at 2 °C/min. The emitter transducer sends ultrasonic waves that travel through the sample until they reach the receiver transducer with a smaller amplitude, and after a defined time referred as time of flight [44]. The difference in amplitude is due to the attenuation of ultrasound propagating through the sample, depending on the material damping capacity, generated by molecular arrangements, macroscopic defects, scattering, and so on. The difference in time is due to the elastic properties and density of the investigated material. The received signals are displayed as echoes. The difference in time and amplitude between emitted and received signal was used to calculate the sound velocity and attenuation by means of an ultrasonic software developed in Labview.

Chemical characterization of EG samples, extracted from composites by dissolution and filtration, was done by X-ray photoelectron spectroscopy (XPS) performed in a K-Alpha equipment by Thermo Scientific (Waltham, MA, USA) with an Al X-ray source. Spectra were calibrated by using the C1s photoemission at 284.6 eV. The beam size used was 400 μm. The energy resolution was 0.10 eV.

## 3. Results

### 3.1. RamanSpectroscopy for EG

**Figure 3:**
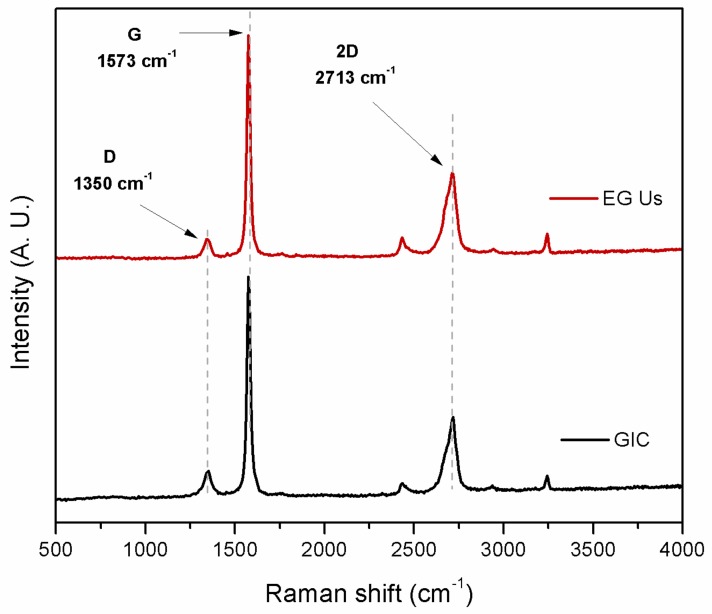
Raman spectra for GIC flakes and EG obtained after ultrasound treatment.

Raman spectroscopy was performed on graphite nanostructures used in this study in order to determine the crystalline quality. The Raman spectra for GIC flakes and EG obtained after ultrasound treatment in water (EG-Us) are shown in Figure 3. Spectra of graphitic materials are characterized by a D band (1350 cm^−1^), a G band (1573 cm^−1^), and a 2D band (2713 cm^−1^). The D band was related to structural disorders and defects by sp^3^ hybridization. The G band was related to stretching vibrations of sp^2^ hybridized carbon atoms (C–C), whereas the 2D band was related to the stacked graphene layers. It is known that the thickness of the graphite nanostructure is reflected in the shape of its 2D band. Graphite nanostructures with more than 10 graphene layers present a broad2D band, which is strongly asymmetric [45].

The I_D_/I_G_ ratio (intensity of the D peak to the intensity of the G peak) was related to the number of defects present in the material. From Raman spectra, we can observe that the D band intensity was smaller than the G band intensity, which indicates that the EG obtained after ultrasonic treatment in water had a high structural quality [46].

### 3.2. Differential Scanning Calorimetry (DSC) Results

**Figure 4:**
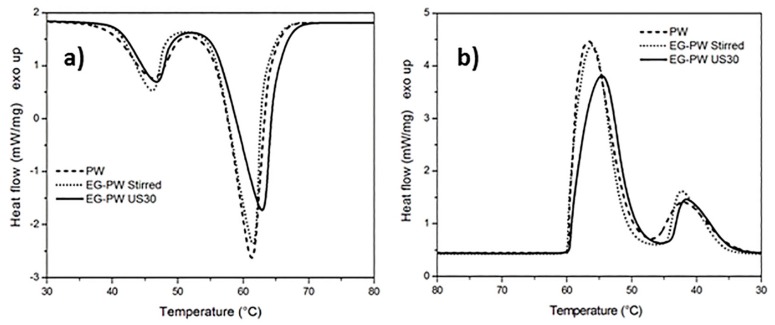
Dynamic differential scanning calorimetry (DSC)thermograms of paraffin wax (PW)–EG during: (**a**) heating and (**b**) cooling at 10 °C/min.

The effect of EG stacks and ultrasonic treatment on the melting and cooling of paraffin and natural wax has been analyzed by means of differential scanning calorimetry (DSC). The dynamic DSC thermograms of pure paraffin PW and EG–PW samples are reported in Figure 4. The DSC heating curves related to pure paraffin (Figure 4a) presented two endothermic phase transitions. As reported in the literature, the lower peak centered at 46–44 °C was ascribed to the solid−solid transition of PW, while the main peak at 61–62 °Cwasrelated to the solid−liquid phase transition of PW [47,48,49,50]. The thermal properties of EG–PW samples are very close to those of pure PW. The addition of EG to PW by simple stirring did not seem to have a significant effect on the melting behavior of paraffin. However, ultrasonic treatment for 30 min led to a slight increase in temperature of the endothermic peak and reduced the melting enthalpy, as reported in Table 1. Similar considerations can be inferred from the DSC thermograms obtained with PW and EG–PW samples during cooling. The enthalpies of melting and crystallization, reported in Table 1, show a considerable change, as also reported by other authors for EG–PW samples not subjected to ultrasonic treatment [49]. This result can be explained assuming that EG behaves as a heterogeneous nucleation agent, leading to a higher number of smaller low-melting crystals [49].

**Figure 5:**
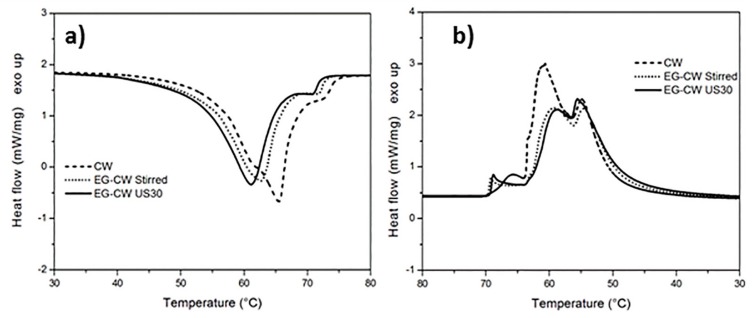
Dynamic DSC thermograms of candelilla wax (CW)–EG systems during: (**a**) heating and (**b**) cooling at 10 °C/min.

The thermogram of pure candelilla wax (Figure 5a) presented a main melting endotherm centered at 65 °C, with a shoulder at around 61 °C followed by a second small peak at 72 °C. The addition of EG and the application of the ultrasonic treatment caused a significant change in the melting behavior of the candelilla wax with a decrease of the temperature of the melting peak at 61 °C. This shift of the melting peak towards lower temperatures indicated the formation of smaller and less stable crystals. This result can be explained, analogous to what was reported above, by the crystallization-promoting effect caused by EG, which behaves as a heterogeneous nucleation center during the crystallization process, as observed in the literature for a different EG–paraffin mixtures [8]. As shown in Figure 5b, the reduced crystallinity in the EG–CW samples could be ascribed to a possible confinement of the chain segments (intercalation), which hindered the segmental rearrangement during crystallization and restricted the formation of perfect crystals in the polymer matrix. A similar loss of crystallinity has been observed in the literature in polymer clay nanocomposites, for example in polyethylene glycol-montmorillonite [51], in Poly(ε-caprolactone)-clay nanocomposites [52], and many others [53]. However, this is the first time that this effect has been demonstrated in the case of candelilla wax mixed with expanded graphite.

The crystallization enthalpy reported in Table 2 confirms that the addition of EG to wax and the use of the ultrasonic treatment led to a reduced crystallinity, probably due to restrictions of the molecular movements. Ultrasonic treatment caused a reduction in the enthalpy of crystallization, from 154.4 to 135.2 J/g for PW-based samples and from 170.4 to 139.9 J/g for CW-based samples (Table 2).

### 3.3. Thermogravimetric Analysis (TGA) Results

**Figure 6:**
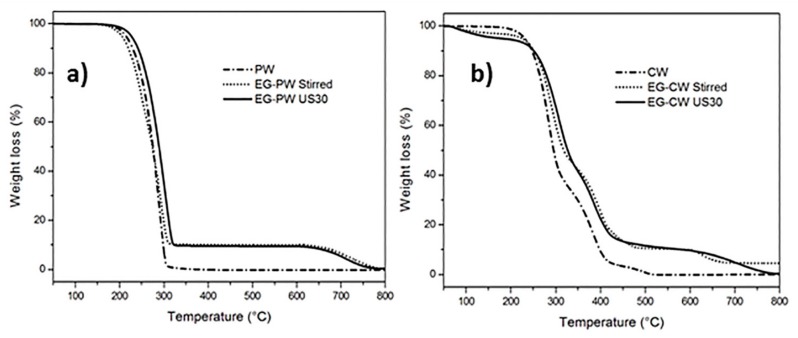
Thermogravimetric analysis (TGA) thermograms of PW–EG and CW–EG systems during heating at 10 °C/min.

The TGA thermograms of pure paraffin and candelilla and their mixtures with expanded graphite are reported in Figure 6. The residual weight at 550 °C and the temperatures of the first (T_DTG1_) and second (T_DTG2)_ peak of the first derivative of the weight loss curve (DTG), are reported in Table 2. Pure paraffin presented a single degradation path, ascribed to the degradation of the paraffin aliphatic chains, where the maximum degradation rate occurred at around 291 °C, as calculated by the peak temperature of the derivative curve. Above 320 °C, the sample completely degraded. Paraffin samples filled with EG showed two thermal degradation steps corresponding to the paraffin degradation and degradation of graphite stacks at 700 °C, associated with the change of purging gas from nitrogen to air at 600 °C. This last weight loss represents 10% by weight of the sample, as expected. The increase of 16 °C in the first degradation temperature (T_DTG1_) for ultrasonic-treated samples indicates that the addition of expanded graphite improved the thermal stability of paraffin.

The TGA thermogram of pure candelilla presented multiple thermal degradation steps, which can be attributed to the molecular weight distribution of the wax, as was confirmed by the results of the gel permeation chromatography in a previous report [13], where a weight distribution with three molecular weight populations was found for pure CW. The CW samples mixed with EG presented additional degradation steps. The first decomposition step at 175 °C (with a weight loss of 3–4%) could be attributed to the presence of volatile compounds and possible residues of D-limonene. The second decomposition at 280 °C can be possibly attributed to the decomposition of the aliphatic chains of the wax. The third decomposition step at 360 °C can be due to the degradation of the polar fractions that decomposed at higher temperatures, and the fourth stage at 650 °C could be attributed to the decomposition of EG in an oxidative atmosphere. In addition, the CW systems with EG presented an increase in the temperature of thermal degradation, with an increase of 24 °C of T_DTG1_ of the EG–CW US30 sample compared to pure CW. This increase of the thermal stability can be attributed to polar or chemical interactions between the CW fractions and the graphite stacks, which could be due to multiple concomitant causes: (i) the polar groups present in the composition of candelilla wax, (ii) the use of D-limonene to prepare the mixtures, and (iii) the ultrasonic treatment, which can lead to chain cleavages, vacancies, and formation of free radicals in the polymeric matrix [54]. This latter cause can promote physical or chemical links with EG.

### 3.4. Scanning Electron Microscopy (SEM) Results

**Figure 7:**
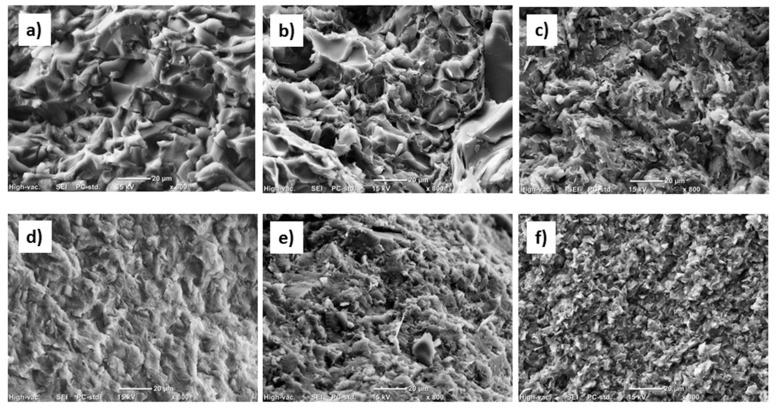
Scanning electron microscopy (SEM) images of investigated samples:(**a**) PW; (**b**) EG–PW Stirred; (**c**) EG–PW US30; (**d**) CW; (**e**) EG–CW Stirred; (**f**) EG–CW US30.

The SEM micrographs of fracture surfaces reported in Figure 7 evidenced a different morphology between pure PW and CW wax. Compared to pure CW (Figure 7d), neat PW (Figure 7a) presented brittle fractures that had a rougher morphology with smaller smooth areas. Candelilla wax presented a morphology characteristic to ductile materials. These differences in morphology were attributed to the molecular composition of waxes. It is known that a ductile fracture in polymers is favored for broad molecular weight distributions, where low molecular weight chains act as plasticizers during fracture. As mentioned above, the molecular weight distribution of candelilla wax was broader than paraffin wax. The incorporation of EG in both waxes led to a change of the morphology that was further modified by ultrasonic treatment, which produced a significant enhancement in rugosity Figure 7c,f). The presence of smaller crystals, as evidenced by DSC analyses in CW-based samples compared to PW ones, was reflected in a rougher fracture surface.

However, the morphology of EG–CW samples was more homogenous than that of EG–PW samples, which was further indicative of better nanofiller dispersions related to interactions among the polar groups present in the CW (esters, alcohols, and fatty acids) and the polar groups present in the surfaces of the graphite layers.

### 3.5. X-ray Diffraction (XRD) Results

**Figure 8:**
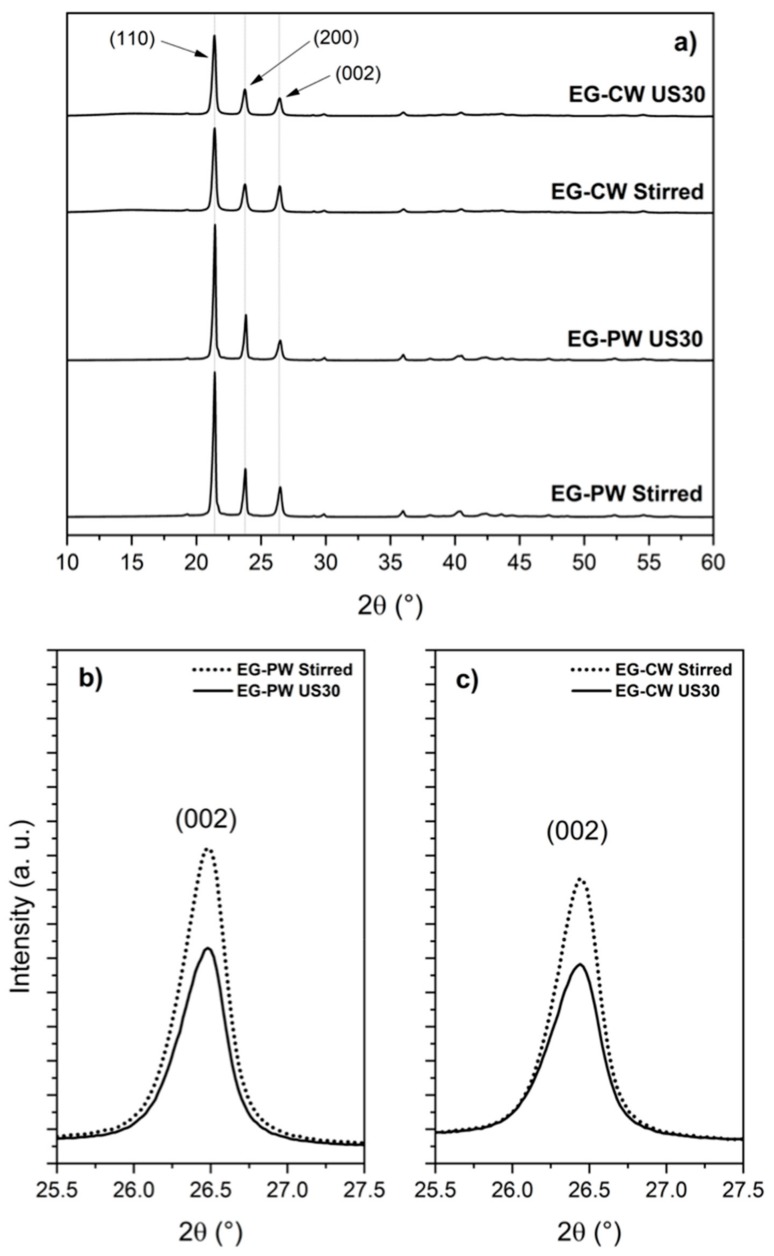
X-ray diffraction(XRD) patterns for wax compounds containing EG. (**a**) XRD patterns showing the crystalline nature of the wax compounds. XRD pattern of the (002) peak of graphite for (**b**) paraffin wax and (**c**) candelilla wax compounds.

An X-ray diffraction analysis (XRD) is commonly used to study the structural properties of graphene nanostructures. Figure 8 shows the XRD patterns for wax compounds, in which the characteristic peaks for these materials are presented. Both wax compounds exhibited two high-intensity X-rays peaks at 21.2° and 23.5° in 2θ, which corresponded to the (110) and (200) diffraction planes, respectively (Figure 8a). These peaks were characteristic of long-chain odd *n*-alkane crystals. In the case of candelilla wax, this crystalline structure was defined by its characteristic component, *n*-hentriacontane (C_31_) [4]. For all wax compounds, the X-ray peak at 26.5° (2θ) confirmed the presence of the graphite structure, corresponding to the (002) diffraction plane. Figure 8b,c shows the intensity of the (002) diffraction plane of graphite considerably decreased with ultrasonic treatment, indicating a partial exfoliation of graphite.

The crystallite size (L_c_) in the direction of the c axis (the average thickness of graphite structure or average stacking height of graphene layers) was determined from the (002) peak using the Scherrer equation:

L_c_ = 0.89λ/βcos(θ), (1)

where λ is the wavelength of the X-rays (1.54183 Å), β is the full width at half maximum (FWHM) of the peak in radians, and θ is the Bragg angle. The results are shown in Table 3. The lowest L_c_ values were obtained for candelilla wax compounds, which revealed that a decreasing thickness of stacked graphene layers was more feasible with this natural wax.

A lower L_c_ value could be achieved with only magnetic stirring in candelilla wax, compared to the system of paraffin wax assisted with ultrasound. This remarkable difference can be mainly attributed to the chemical composition of candelilla wax. As we mentioned above, candelilla wax presents a mixture of compounds different than *n*-alkanes, such as fatty alcohols and fatty acids. These compounds, having polar groups, could interact better with polar groups of the EG, facilitating the exfoliation process.

### 3.6. Rheology

**Figure 9:**
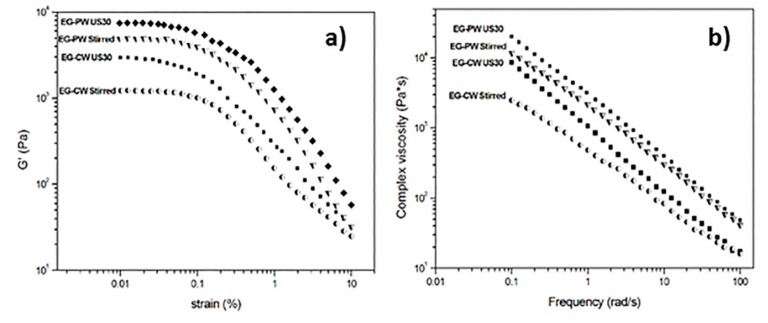
Rheological behavior of EG–wax systems at 80°C: (**a**) dynamic strain sweep at 6.28 rad/s; (**b**) frequency sweep measurements at 0.03% strain.

Melt rheology was used to analyze the dispersion/exfoliation of expanded graphite in wax matrices. The dynamic strain sweep measurements at 80 °C for the dispersions with different wax matrixes and treatments are compared in Figure 9a. A fixed frequency of 6.28 rad/s was kept constant during the experiment while the strain increased. Because of the sensitivity of the load cell of the instrument, reliable data on pure wax could not be obtained. The storage modulus (G’) was at least 10 times higher than the loss modulus (G’’) that, for sake of clarity, was not reported. This indicates that the EG–wax mixtures showed a gel or solid-like behavior in the linear viscoelastic region, probably resulting from the formation of a three-dimensional network involving weak bonding forces between the organic phase and EG. During the strain sweep measurements, the storage modulus G’ remained constant as far as the sample structure was maintained. When the gel intermolecular forces were overcome by the oscillation stress, the sample broke down, and the modulus fell. The maximum strain (γ_crit_), to which the linear viscoelastic region extended, was determined as the value above which G’ decreased more than 10% of the maximum value [55]. As can be clearly observed in Figure 9a and from the γ_crit_ values reported in Table 4, under the same wax matrix, ultrasonic treatment led to a significant increment in the storage modulus and a reduction of the linear viscoelastic region (decrease of γ_crit_).

The frequency sweep curves, reported in Figure 9b, were obtained using a very low strain value (0.025%) within the linear viscoelastic region. For each EG–wax sample, G’ was larger than G’’ in the whole experimental frequency range, showing that the elastic behavior of the dispersions was dominant compared to the viscous one. All samples demonstrate shear-thinning behavior at low frequencies. It is known from literature that the rheological behavior, especially in the linear viscoelastic region, is highly sensitive to the nanostructure developed during processing. For both CW and PW systems, an increase in the complex viscosity was observed for the samples ultrasonically treated. This η* increase is usually considered an indication of an increased degree of exfoliation of the nanofiller.

The shear-thinning region is well fitted by a power law expression:

|η*| = Aω^n^, (2)

where |η*| is the modulus of complex viscosity, A is a sample-specific pre-exponential factor, ω is the oscillation frequency in the frequency sweep test, and n is the shear thinning exponent. A and n can be directly determined from the logarithmic plot of viscosity (η*) versus frequency (ω) as:

log(|η*|) = log A + nlog(ω).(3)

The exponent n, extracted from the linear fit of the low-frequency data, has been proposed by several authors as a semi-quantitative measurement of the degree of nanofiller exfoliation in a polymer matrix [56,57,58]. An increase in the shear thinning exponent n is considered related to an increase in the extent of exfoliation. A value of n close to zero is considered in systems where no exfoliation occurs, and a value close to one corresponds to full exfoliation [59].

The values of shear thinning exponent n are reported in Table 4. For each wax typology, the sonicated sample presented a higher n exponent compared to the stirred sample, which is in agreement with the results of Wang et al.’s [60] montmorillonite-paraffin wax systems. However, this is the first time that a similar comparison between sonicated and stirred nanocomposites is presented for EG–PW and EG–CW systems. The increase of the shear thinning exponent, due to the ultrasonic treatment, was about 11% and 28% for the EG–PW and EG–CW systems, respectively. Moreover, the exponent n for sonicated EG–CW samples was higher than that of EG–PW samples. This is a further indication that the CW nanocomposite samples presented a higher degree of EG dispersion after ultrasonic treatment. In agreement with the DSC, XRD, and SEM results, the rheological results also confirm the potential of natural wax as a dispersing medium for expanded graphite. This opens the way to countless applications of EG–CW dispersions in the nanocomposite field.

### 3.7. Ultrasonic Dynamic Mechanical Analysis (UDMA) Results

**Figure 10:**
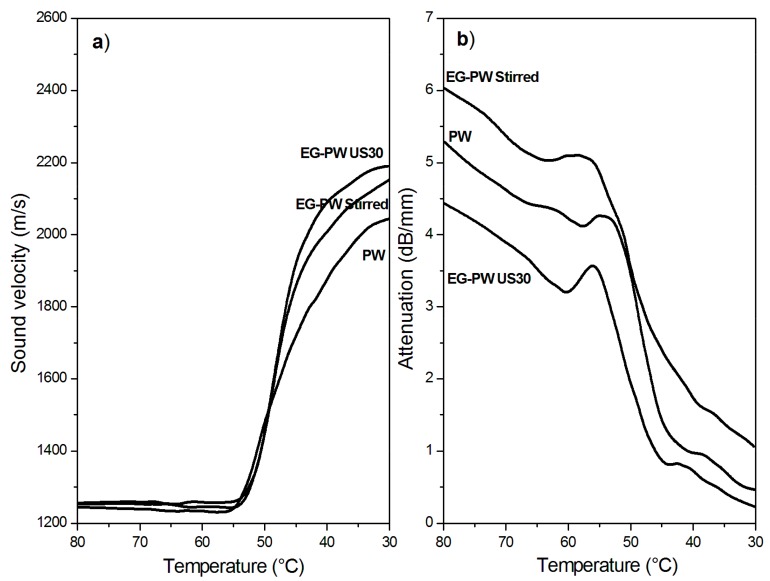
Temperature variation of (**a**) the sound velocity and (**b**) attenuation in neat paraffin wax and EG–PW samples during cooling at 2 °C/min.

**Figure 11:**
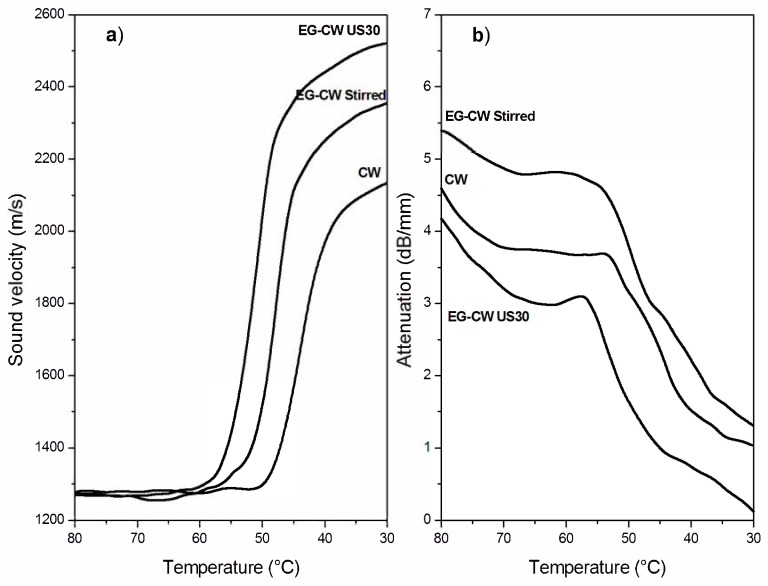
Variation of (**a**) the sound velocity and (**b**) attenuation in neat candelilla wax and EG–CW samples during cooling at 2 °C/min.

Figure 10a and Figure 11a show the temperature dependence of the sound velocity in paraffin and candelilla wax and their nanocomposites with EG during cooling at 2 °C/min from the molten state. The longitudinal velocity curves were characterized by a nonlinear growth that corresponded to the crystallization leading to a progressive sample stiffening with decreasing temperature. The increase in the longitudinal velocity reflected the growth of the elastic response of the sample, which became dominant over the viscous response because of the nucleation and growth of crystals during cooling [61]. The increase in the sound velocity became rapid over a temperature range rather than at a single temperature value since both paraffin and candelilla wax are made of a mixture of hydrocarbons C*_n_*H*_2n+2_* characterized by a broad range of crystallization temperatures (Figure 4b and Figure 5b).

Even if the dependence of the sound velocity in the investigated wax samples is similar, some differences can be observed between the paraffin and candelilla samples. First of all, the sound velocity of molten CW samples was slightly higher (35 m/s) than that of PW samples since it reflects the different molecular composition of the two waxes. Moreover, the temperature corresponding to the increased sound velocity was quite close for all investigated PW samples, while it was different for CW samples. This was in agreement with the DSC thermograms obtained during cooling scans (Figure 4b and Figure 5b). For both PW and CW samples, the presence of EG affected the final velocity value, which was higher in the case of the sonicated samples, indicating the development of higher elastic properties.

The temperature dependence of the sound attenuation in PW and CW samples during cooling at 2 °C/min from the molten state is reported in Figure 10b and Figure 11b. Sound attenuation in a material depends on the viscous behavior and homogeneity of the material, since the sound energy losses result from: (i) molecular absorption, which is related to molecular relaxations in the polymer structure, and (ii) scattering, which can be relevant in non-homogeneous materials containing agglomerates with sizes comparable to the sound wavelength [28]. The attenuation curves in Figure 10b and Figure 11b present an initial decrease with the temperature caused by the reduction of the absorption losses as a consequence of the reduced molecular relaxations. During cooling, in fact, the wax fractions with high molecular weight started to arrange in an ordered way, thus presenting a reduced mobility and, consequently, reduced relaxation phenomena [62]. Upon further cooling, the attenuation increased because of increased scattering losses arising from the growth of crystallites followed by their aggregation. Under crystallization, attenuation reached a maximum, after which it decreased since a more homogenous structure in the solid wax sample formed, thus reducing the scattering losses.

The attenuation of stirred EG–wax samples was higher than that of neat wax samples in the investigated temperature range, thus indicating the presence of some expanded graphite clusters. However, when the nanocomposite samples were sonicated, the attenuation reduced, becoming even lower than that of the neat wax. As reported by Espinoza et al. [28] on the degree of dispersion of carbon nanotubes (CNTs) in nanocomposites treated with high-intensity ultrasound, a low sound attenuation is observed when there is a significant reduction in the size of the agglomerates, being of a size not comparable to US wavelengths. Therefore, the UDMA results are indicative of the reduction of the cluster size and of a better filler dispersion in the wax matrix promoted by the cavitation caused by ultrasonic treatment.

**Figure 12:**
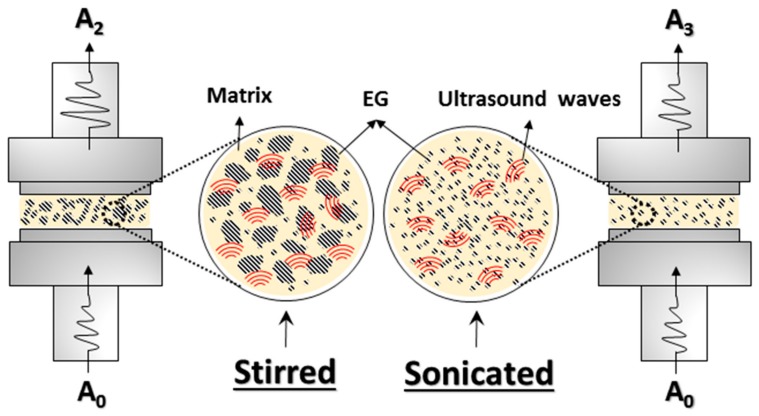
Schematic representation of ultrasonic dynamic mechanical analysis (UDMA) measurements in stirred and sonicated samples.

A sketch of the morphology of stirred and sonicated samples undergoing UDMA measurements is compared in Figure 12. The increase in ultrasonic attenuation was mainly due to the scattering of ultrasonic waves, which resulted from the presence of particles or agglomerates with sizes comparable to the wavelength [63]. When the waves pass through a sample with agglomerates much smaller than the wavelength (less than 25% of the wavelength), scattering does not occur, and a large part of the waves is transmitted while the other part is absorbed, causing a decrease of the initial wave amplitude in the molten sample at 80 °C. So, according to the sketch of Figure 12, the amplitude A_2_ was lower than A_3_ essentially as a consequence of the scattering produced by larger EG agglomerates.

Finally, it can be observed that CW-based samples showed lower attenuation values than PW-based samples. This can be attributed to the higher affinity between the polar groups present in the natural wax and the graphite layers, which improve the steps involved in dispersion (i.e., wetting of initial agglomerates, infiltration of low molecular chains in the graphite layers, and the dispersion of agglomerates). Therefore, UDMA results confirm dispersion improvement using natural wax as a pre-exfoliating medium.

### 3.8. X-ray Photoelectron Spectroscopy (XPS) Results

**Figure 13:**
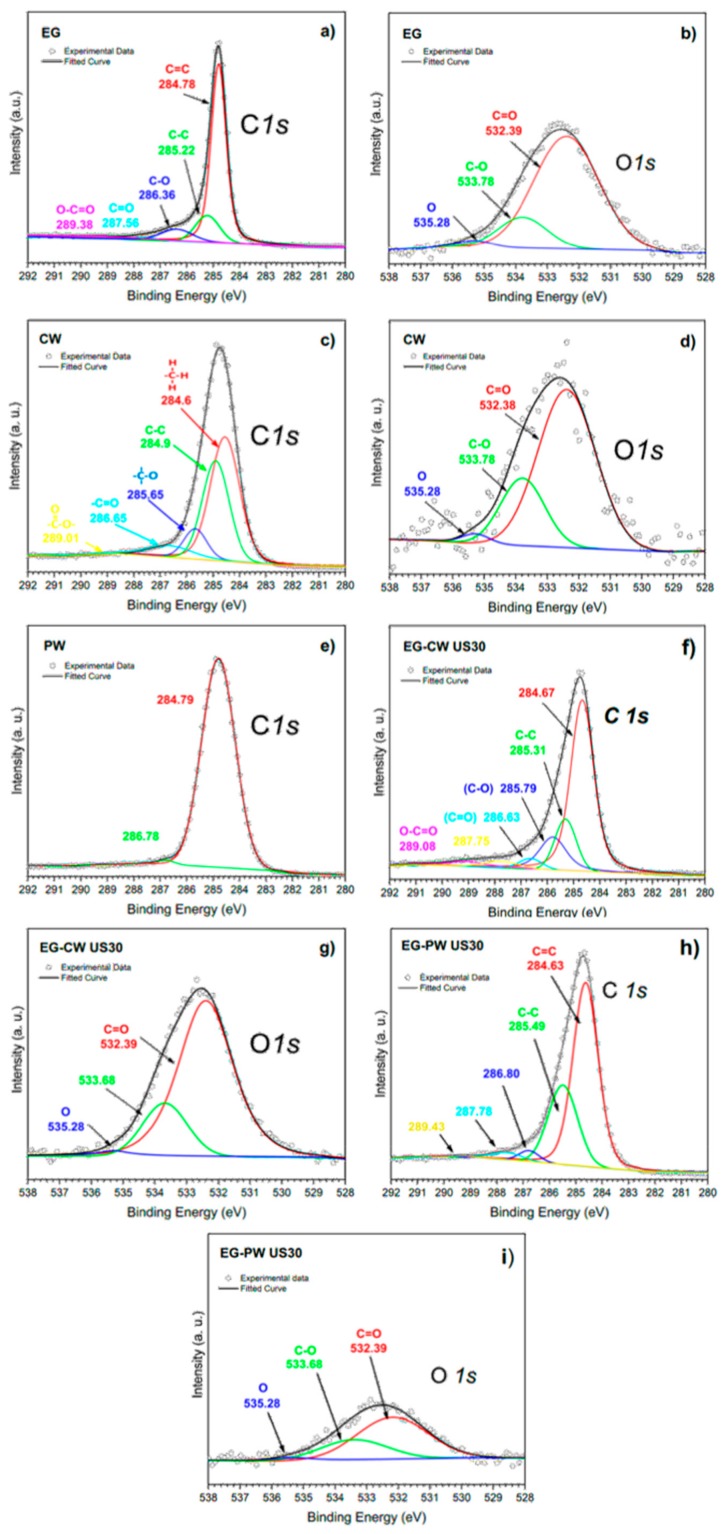
X-ray photoelectron spectra of wax matrices and expanded graphite extracted from ultrasound-treated wax composites. (**a**,**b**) Pristine EG. (**c**,**d**) CW matrix, (**e**) PW matrix, (**f**,**g**) EG-CW ultrasound treated, and (**h**,**i**) EG-PW ultrasound treated.

The presence of interactions between the polar groups present in the natural wax and the polar groups at the surface of expanded graphite layers was also confirmed by X-ray photoelectron spectroscopy (XPS), as reported in Figure 13. To identify every peak in the spectral set, NIST (National Institute of Standards and Technology) XPS Database 20, Version 4.1 was used. The carbon (C1s) spectrum for EG was deconvoluted into five components (Figure 13a). In detail, the main peak at 284.78 eV was associated with photoelectrons emitted from sp^2^-hybridized carbons (C=C) in aromatic rings of the graphene lattice. The component with binding energy of 285.22 eV was assigned to sp^3^carbon–carbon single bonds (C–C), which are attributed to structural defects derived upon ultrasonication [64]. The components with binding energies of 286.36 and 287.56eV are typically assigned to the C–OH and C=O functional groups, respectively [65].

The binding energies around 286.50 eV can also include ether or epoxide groups (C–O–C), which have a similar binding energy to C–OH [66].On the other hand, the fifth component at 289.38 eV was attributed to carbon atoms in carboxylic groups (O=C–O) [64].The presence of oxygen functional groups on EG was due to thermal and ultrasonic treatments that induce oxygenated species onto the graphene lattice [64].The components in the C1s spectrum for EG, the O1s peak at 532.39 eV, were assigned to contributions from C=O and O=C–OH groups and that at 533.78 eV to C–OH groups (Figure 13b). Table 5 shows the relative area of functional groups found for EG samples, which were calculated from C1s XPS spectra. Neat EG presented a carbon percentage of 89.91% and had a very high oxygen percentage (~10.09%), which is similar to those values reached in systems under ultrasonic treatment in the presence of polar solvents [64]. Due to structural defects generated on the graphene lattice during sonication, different oxygen-containing species can react on these active sites. When water is used as solvent, these oxygen-containing species can be derived from hydroxyl radicals (OH⋅) generated from sonolysis of water [67]. Other sources of oxygen to be considered are HO_2_⋅ radicals, which are produced from reactions between hydrogen atoms and O_2_, soluble in water, generated during sonolysis.

The C1s and O1s XPS spectra for candelilla wax are presented in Figure 13c,d for the first time in the literature. The C1s XPS spectrum was fit with up to five peaks using binding energies of 284.60, 284.90, 285.65, 286.65, and 289.01 eV, which can be attributed to C–H, C–C, C–O, C=O, and O=C–OH groups, respectively. The C–H and C–C groups were assigned to long-chain aliphatic hydrocarbons, whereas oxygen-containing groups were assigned to species such as wax esters, acid esters, secondary alcohols in even-numbered carbon chains (C28 to C34), free acids, free alcohols, and sterols [5]. Therefore, by reference to the components in the C1s spectrum, the O1s peaks were assigned to contributions from these oxygen-containing species. In contrast, the C1s spectrum for paraffin wax (Figure 13e) presented a main peak centered at 284.50–285.50 eV, which covered binding energies of the C–H and C–C groups assigned to long-chain aliphatic hydrocarbons. It was not possible to obtain the O 1s spectrum for PW since PW does not have oxygen groups.

On the other hand, Figure 13f–i presents the C1*s* and O1s XPS spectra for EG samples extracted from ultrasound-treated wax composites. The XPS data show that binding energies for components present at C1s and O1s spectra were similar to those obtained for neat EG. However, significant differences were found in the relative area percentages of functional groups (Table 5) since EG extracted from candelilla wax presented a very high percentage of oxygen-containing species (~18.18%) compared to that obtained from paraffin wax (~9.13%). The increase in the oxygen percentage suggests that compounds with oxygen-containing functional groups in candelilla wax were grafted on surface of graphene lattices. It has been reported that ultrasonic treatment of oligomers and polymers generate the rupture of chemical bonds, promoting the grafting of molecular chains on carbon nanostructures [21]. However, this phenomenon has not been reported using natural waxes.

The most relevant result on XPS is focused on evidence of molecular chains of wax grafted on graphite layers, which are more predominant for CW composites. A plausible explanation relies on an abundance of molecular chains of wax with more functional groups than paraffin, which could have the ability to react with functional groups of EG under ultrasonic irradiation. These grafted groups improve the exfoliation-dispersion of graphite layers, reduce the density of agglomerate, which would facilitate the formation of a relatively stable suspension, and modify some thermal properties of concentrates.

## 4. Conclusions

In this work, multiple techniques have been applied for studying the dispersion of a high amount (10% by weight) of expanded graphite in natural wax (candelilla) obtained by a novel, green approach involving high-power ultrasound and an organic natural solvent, D-limonene. The following conclusions can be drawn:

1. DSC analysis suggests that the reduced crystallinity in the EG–CW samples can be ascribed to a possible confinement of the chain segments (i.e., intercalation), which hinders the segmental rearrangement during crystallization and restricts the formation of perfect crystals in the polymer matrix.
2. TGA indicates that the addition of expanded graphite improves the thermal stability of paraffin and candelilla wax, probably due to the interactions between the matrices and the graphite stacks.
3. XRD analysis revealed that the ultrasonic treatment of EG in candelilla wax induces a higher exfoliation of graphite, demonstrated by a considerable decrease in the intensity of the (002) diffraction plane of graphite. Polar groups present in candelilla wax compounds could interact better with polar groups of the EG, facilitating the exfoliation process.
4. Rheological analysis by dynamic oscillatory rheometry has proved that EG–wax mixtures show a gel or solid-like behavior in the linear viscoelastic region, probably resulting from the formation of a three-dimensional network (i.e., a physical gel) involving weak bonding forces between the organic and inorganic phase. Ultrasonic treatment leads to a significant increment in the storage modulus, a reduction of the linear viscoelastic region, and an increase of the shear thinning exponent. This is a further indication that the CW nanocomposite samples present a higher degree of EG dispersion after ultrasonic treatment.
5. UDMA results are indicative of the reduction of the cluster size and of a better filler dispersion in the wax matrix promoted by the cavitation caused by ultrasonic treatment.
6. XPS analysis indicates that the ultrasonic treatment promotes the grafting of CW compounds with oxygen-containing functional groups on the surface of graphite layers, due to the chemical composition of the natural wax. These grafted groups improve the exfoliation-dispersion of graphite layers, facilitate the formation of a relatively stable suspension, and modify some thermal properties of natural concentrates.

In a future work, the developed CW–EG masterbatch, containing 10% by weight of EG, will be used to promote the dispersion of EG in non-polar polymers.

## 5. Patents

Carlos Espinoza-González, Oliverio Rodríguez-Fernández, Lidia Delgado-Interial, Salvador Fernández-Tavizón, and Layza Arizmendi-Galaviz; “Latent heat storage composites and method therefor” MX/a/2018/008923.

## Figures and Tables

**Table 1:** Thermal properties for the PW- and CW-based samples.

Sample	ΔH_m_ (J/g)	T_m_ (°C)	ΔH_c_ (J/g)	T_c_ (°C)
PW	148.1	61.3	154.4	56.6
EG–PW Stirred	142.3	61.5	147.1	56.1
EG–PW US30	129.1	62.9	135.2	54.6
CW	167.4	65.5	170.4	65.7 / 60.8 / 54.8
EG–CW Stirred	142.1	62.6	135.5	69.2 / 59.3 / 54.5
EG–CW US30	144.4	61.1	139.8	68.9 / 58.7 / 55.5

**Table 2:** TGA results for the CW and PW samples.

Sample	Residual Weight at 550 °C (%)	T_DTG1_ (°C)	T_DTG2_ (°C)
PW	0.05	291.2	-
EG–PW Stirred	10.1	295.7	-
EG–PW US30	10.4	307.2	-
CW	0.03	285.6	381
EG–CW Stirred	10.3	291.6	396
EG–CW US30	10.2	309.9	392

**Table 3:** Results of crystallite size for wax compounds.

Sample	Angle 2θ (°)	d_002_ (Å)	FWHM (°)	Lc (Å)
PW–EGStirred	26.45	3.36	0.221	385 ± 20
PW–EG Us-3030	26.40	3.37	0.267	319 ± 6
CW–EGStirred	26.46	3.36	0.326	262 ± 3
CW–EGUs-3030	26.45	3.36	0.374	228 ± 2

**Table 4:** Rheological parameters for the PW- and CW-based samples.

Sample	γ_crit_ (%)	n -	R^2^
EG–PW Stirred	0.057	0.73	0.998
EG–PW US30	0.045	0.81	0.999
EG–CW Stirred	0.070	0.71	0.999
EG–CW US30	0.034	0.91	0.999

**Table 5:** Relative area percentages of functional groups found for EG samples and calculated from C1s XPS spectra.

Sample	Signal	Assignment	Binding Energy (eV)	Peak Area (%)
**EG**	C1s	C=C	284.78	73.55
		C-C	285.22	16.36
		C-O	286.36	8.86
		C=O	287.56	1.24
**EG–PW US30**	C1s	C=C	284.63	60.67
		C-C	285.49	30.20
		C-O	286.77	2.84
		C=O	287.62	6.29
**EG–CW US30**	C1s	C=C	284.67	64.37
		C-C	285.31	17.45
		C-O	285.79	12.70
		C=O	286.63	3.73
		Other		1.75
